# An End-to-End Steel Surface Classification Approach Based on EDCGAN and MobileNet V2

**DOI:** 10.3390/s23041953

**Published:** 2023-02-09

**Authors:** Ge Jin, Yanghe Liu, Peiliang Qin, Rongjing Hong, Tingting Xu, Guoyu Lu

**Affiliations:** 1 School of Mechanical and Power Engineering, Nanjing Tech University, Nanjing 211816, China; 2 Intelligent Vision and Sensing Lab, University of Georgia, GA 30602, USA; 3 Changchun Institute of Optics, Fine Mechanics and Physics, Chinese Academy of Sciences, Changchun 130033, China

**Keywords:** defect classification, image processing, generative adversarial networks, data augmentation, multi-training, deep learning

## Abstract

In the production process of steel products, it is very important to find defects, which can not only reduce the failure rate of industrial production but also can reduce economic losses. All deep learning-based methods need many labeled samples for training. However, in the industrial field, there is a lack of sufficient training samples, especially in steel surface defects. It is almost impossible to collect enough samples that can be used for training. To solve this kind of problem, different from traditional data enhancement methods, this paper constructed a data enhancement model dependent on GAN, using our designed EDCGAN to generate abundant samples that can be used for training. Finally, we mixed different proportions of the generated samples with the original samples and tested them through the MobileNet V2 classification model. The test results showed that if we added the samples generated by EDCGAN to the original samples, the classification results would gradually improve. When the ratio reaches 80%, the overall classification result reaches the highest, achieving an accuracy rate of more than 99%. The experimental process proves the effectiveness of this method and can improve the quality of steel processing.

## 1. Introduction

In industrial production, defect classification is an essential step in the steel manufacturing process. However, this step is usually performed manually and mainly relies on the human eyes to observe defects on the steel surface. Therefore, production efficiency and product quality are generally low. The manual detection method is difficult to guarantee the detection effect. Even using human eyes to find defects for a long time may cause damage to human health. So, automatically detecting defects to replace manual labor in industrial manufacturing has become an important research direction [1,2].

In the industrial manufacturing environment, there are many difficulties in steel surface defect detection, such as slight differences, low contrast, too many noises, etc. [3]. The methods based on traditional machine learning are generally to preprocess the collected images, feature extraction, then use a classifier to determine different types of defects. The classifier needs to extract characteristics from the steel surfaces figure. Widely used features include LBP [4], Haar [5], HOG [6], multi-scale analysis, etc. For example, S. R. Aghdam proposed a method to detect steel surface defects based on decision trees [7]. S. Ghorai et al. use a three-level Haar feature to design an automatic detection algorithm applied to production defects on the surface of steel [8]. Shumin D et al. propose a traditional model, which is based on the SVM method, for detecting fabric defects [9]. Although these methods can reduce manual labor, the features used by the classifier still need to be designed manually. Moreover, these detection results using traditional machine learning methods still have obvious deficiencies.

Recently, deep learning technology has exhibited excellent performance in many fields of AI. This method builds multiple layers of neural network models, to continuously adjust model parameters in the training process, and finally fits the task [10]. There are many methods for defect classification on the mental surface. For instance, Fu et al. designed an end-to-end SqueezeNet-based model for this kind of classification [11]. Z. Huang et al. use a DARCNN, which was applied to classify hot-rolled steel strip defects [12]. Li J et al. propose a method for real-time classification according to the model of deep CNN [13]. Tao X et al. propose a novel cascaded autoencoder (CASAE) architecture that successfully detects metal defects under various conditions [14]. He Y et al. propose a novel defect detection system and focus on steel plate defect detection [15]. Li M et al. propose a detection technology for steel strip surface defects according to the improved YOLO algorithm, embed the attention mechanism in the model, modify the path aggregation network, and strengthen the feature extraction performance of the model [16].

Although these methods design various deep learning models, their results mainly rely on the supervised training process. For the shortage of large, labeled defect samples, they have to use networks with few layers, which are usually not deep enough and have no solid, expressive ability. The insufficient labeled training samples also lead to the risk of overfitting, resulting in this trained network model seeming to be less robust when testing in the natural environment. The deep learning network with insufficient labeled training samples is a problem in training deep networks and may seriously affect the expressiveness of the model. Therefore, for the task of supervised learning, a sufficient training dataset is essential [17]. 

The training of such kinds of network models needs a large enough labeled dataset. Yet unlike life scenes, the defect samples in the industrial field are very few. Therefore, using data augmentation to expand the training data set is a strategy to solve the insufficient training dataset [18,19]. We design an EDCGAN to generate massive training labels to solve the problem of insufficient training datasets. When there are enough training samples, the deep network model can be better trained, thereby enhancing the model’s precision. Finally, we use experiments to compare data sets with different ratios and other classification algorithms. The results prove that our method can effectively solve the problem of insufficient training data samples of steel surface defects and improve the accuracy of classification.

## 2. Related Work

### 2.1. Generative Adversarial Network

**Figure 1:**
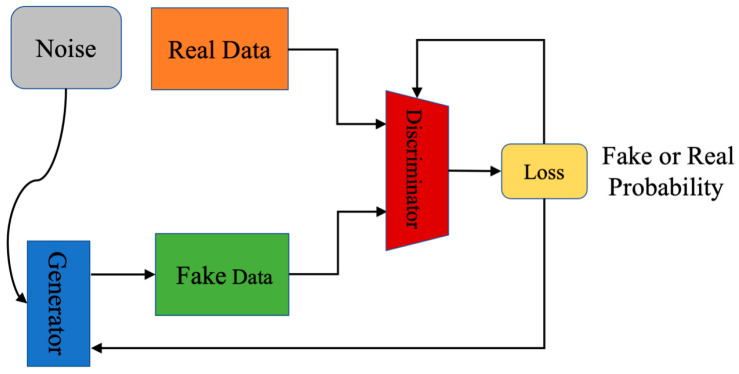
The structure of the standard GAN.

The basic principle of a generative adversarial network (GAN) is that a generative model (G) generates fake samples through the input of random noises, then input these generated samples into a discriminant model (D) and uses the discriminant model to judge the origin of samples. By adversarial training of G and D, the samples generated based on the generative model are closer and closer to real samples, and the ability of the discriminator is also getting stronger and stronger. The network model of standard GAN can be seen in Figure 1. 

GAN uses the value function of Formula (1) to play a two-player minimax game. $p_{z}\left(z\right)$ is the input noise variables, $D\left(x\right)$ represents the probability value of X coming from the dataset. The training task of the model is to use *D* to maximize the probability of calculating the correct label for the input samples, and train *G* to minimize the value of $\log\;\left(1-D\left(G\left(z\right)\right)\right)$.

(1) $$\underset{G}{\min}\;\underset{D}{\max}\left(G,D\right)=E_{p_{data}\left(x\right)}\log_{\;}Dx+E_{p_{z}\left(𝓏\right)}\log\left[1-D\left(G\left(𝓏\right)\right)\right]$$

During this process, the *G* and the discriminator (*D*) are optimized simultaneously. For the *G*, it is hoped that the image it generates is as closer to the real image as possible. The optimization function of the *G* is shown as Formula (2); the smaller its value is, the closer the generated image is to the real sample.

(2) $$\nabla_{\theta_{d}}\frac{1}{m}\sum_{i=1}^{m}\left[\log\left(1-D(G({𝓏}^{\left(i\right)}))\right)\right]$$

At the same time, it is hoped that the *D* can correctly distinguish the true or false of the input images; its optimization function is shown in Formula (3). The value is positive correlated to the discriminator model property.

(3) $$\nabla_{\theta_{d}}\frac{1}{m}\sum_{i=1}^{m}\left[\log D\left(x^{i}\right)+\log\left(1-D(G({𝓏}^{\left(i\right)}))\right)\right]$$

These above two functions are optimized repeatedly and alternately. Finally, the performance of the generator and discriminator reaches a balanced state that the generator cannot generate more realistic samples and the discriminator cannot correctly identify different samples.

### 2.2. Classification Algorithms

The advantage of the classifier based on deep learning is that it can efficiently learn more abstract features in the training process; without the need to design targeted artificial features. The classification accuracy is obviously improved. Convolutional neural network (CNN) is widely used in the field of surface classification due to their various performance advantages. CNN needs to be guided in the training process. It shows the characteristics of local connection, weight sharing, etc., which greatly reduces the number of parameters and the difficulty of model training. It can efficiently learn local data features and has good stability after various linear transformations of the image. In this process, the deep network model is mainly used to convolve; for nonlinear activation function mapping, and as a pooling layer to obtain the mapping relationship between variables. The structure of the CNN model is mainly composed of multiple convolutional layers and pooling layers with different functions. In the field of image classification, the classification network model using CNN has been widely used. Common classification algorithms are VGG [20], AlexNet [21], ResNet [22], MobileNet [23], and SENet [24].

### 2.3. MobileNet V2

**Figure 2:**
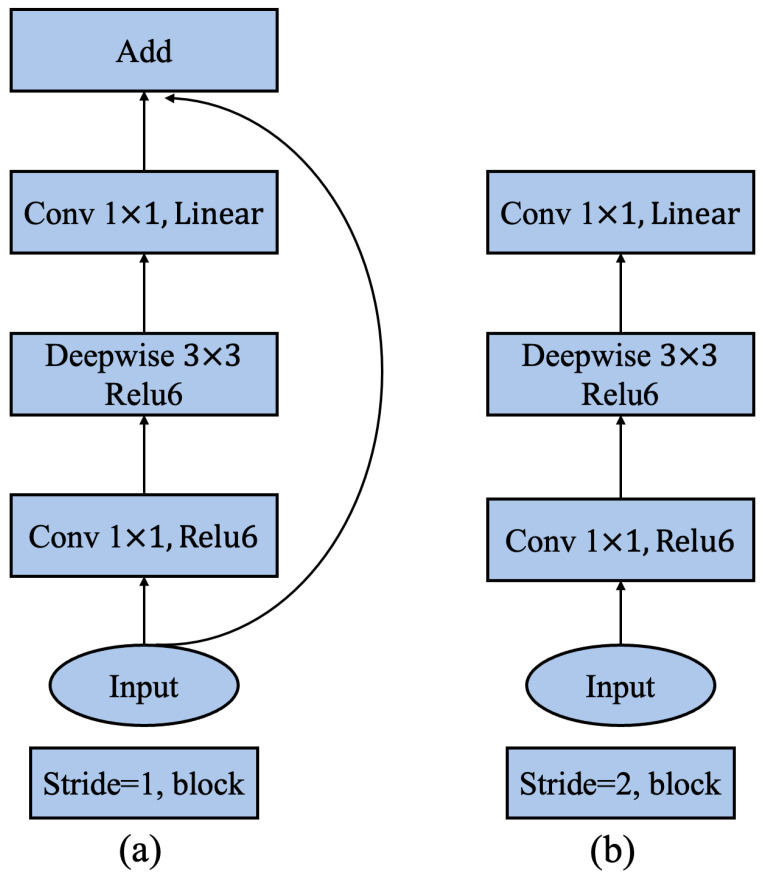
MobileNet V2 solves the degradation issue associated with the increase in the number of network layers by introducing the inverted residual structure and the shortcut. (**a**) Is the residual block when the step size is 1, and (**b**) is the residual block when the step size is 2.

CNN has been applied in the task of target classification; to pursue better accuracy, the depth of these models is getting deeper and deeper, and the models are getting more and more complex. However, when these models are used in real production, problems often happen. These models require powerful equipment and network support, but it is usually unavailable in the industrial field. Therefore, some network models redesign the structure to optimize the model in terms of speed and complexity. MobileNet V2 is one of these representatives; it is a lightweight deep neural network model built using depthwise separable convolutions that can effectively extract data space and channel features by equivalenting the general convolution method into two newly defined convolution operations. Additionally, two global hyperparameters are referenced in MobileNet V2, namely width multiplier: α and resolution multiplier: ρ to balance delay and precision. The basic unit of MobileNet V2 is the depthwise separable convolution. The most important improvements of MobileNet V2 are the inverted residual structure and linear bottlenecks. The inverted residual structure is the modules connected to the residuals. First, the projection convolution is used to increase the dimension, then the depth convolution is used, and finally, the projection convolution is used to reduce the dimension. The combined structure of inverted residual structure and linear bottlenecks is shown in Figure 2.

Compared with traditional high-performance network models, the number of parameters of MobileNet V2 has been greatly optimized, while the model’s performance is not significantly degraded. Hence, it is a lightweight network model that can ensure high accuracy. Moreover, it can be easily and quickly deployed on the mobile terminal and can even perform inference without network communication, so it is suitable for industrial fields with complex environments.

For an input feature map, its size is, where the feature size is $D_{f}\times D_{f}$. If the output feature map size is $D_{g}\times D_{g}\times N$, the size of its convolution kernel is $D_{k}\times D_{k}\times M$, the number of parameters of that is $D_{k}\times D_{k}\times M\times N$, and the calculation amount of the model is $D_{k}\times D_{k}\times M\times N\times D_{f}\times D_{f}$. The convolution process is into two steps. The total computational cost of training the model is $D_{f}\times D_{f}\times 1$, the convolution kernel of pointwise convolution is 1×1×M, and the calculation amount of the model is $\left(D_{f}\times D_{f}\times M\right)\times M\times D_{f}\times D_{f}$. Therefore, the calculation ratio of MobileNet V2 convolution and ordinary convolution is as follows:

(4) $$\frac{D_{\kappa }\times D_{\kappa }\times M\times D_{f}\times D_{f}+M\times N\times D_{f}\times D_{f}}{D_{\kappa }\times D_{\kappa }\times M\times N\times D_{f}\times D_{\kappa }}=\frac{1}{N}+D_{\kappa }^{2}$$

For the general 3×3 convolution kernel, the calculation amount of MobileNet V2 is 1/9 of the calculation amount of the ordinary convolution method. Compared with MobileNet V1, it greatly reduces practical loss without reducing performance by optimizing the structure. This paper applied MobileNet V2 as the base network to implement the classification of steel surfaces. 

## 3. Network Architecture

### 3.1. The Network Architecture of EDCGAN

DCGAN (deep convolution GAN) is a network model with deep convolutional layers, which adds the CNN layers to the GAN network. It is an improvement in the network architecture of the original GAN model. In DCGAN, both the generator and discriminator use CNN architecture; and can be properly optimized according to application requirements. The discriminator of DCGAN retains the architecture of CNN, while the generator applies fractional step convolution, which can better meet the performance-related requirements. 

Compared with other GAN models, DCGAN can flexibly choose the activation function and loss function, and different choices can directly affect the training results of the network model. Without the activation function, the output value can only be a linear expression of the input value, and the school performance of this model is very poor. The introduction of a nonlinear activation function can significantly improve the performance of the DNN network. It can accurately approximate any function, which is of great significance in improving the performance of the model. 

Activation function can directly affect the high-dimensional mapping ability from noises to meaningful images, so different activation functions can greatly affect the quality of generated images. In the original DCGAN model, batch normalization is applied after the activation function layer. However, batch normalization needs to calculate the variance and offset of the data distribution. As the network layers get deeper and deeper, more and more batch normalizations need to be performed, which leads to an increase in calculation during the training process. 

To enable the DCGAN model to generate better quality images, this paper constructed an EDCGAN model that uses the ELU function to replace the ReLU activation function in the original DCGAN. ELU activation function can not only generate a nonlinear relationship between the input and output but also can automatically normalize the input neuron data, eliminating the need for adding batch normalization in the DCGAN structure and reducing the computational complexity of neural network training. ELU is an improved version of ReLU; unlike the sparsity of the ReLU activation function, ELU can provide richer features as it preserves computations with less than 0 inputs; its activation function is shown in Formula (5). ELU also has the property of self-normalization, which can keep the output of this neuron self-normalized. Normalization of parameters is essential for neural networks. If there is no normalization process, the data distribution in a certain network layer will likely have a certain offset phenomenon. As the number of network layers increases, this problem will aggravate, leading to increased difficulty in model optimization; or even impossible optimization.

(5) $$f\left(x\right)=\left\{\begin{array}{ccc} x & , & x>0\\ \alpha \left(e^{x}-1\right) & , & x\leq 0 \end{array}\right.$$

**Figure 3:**
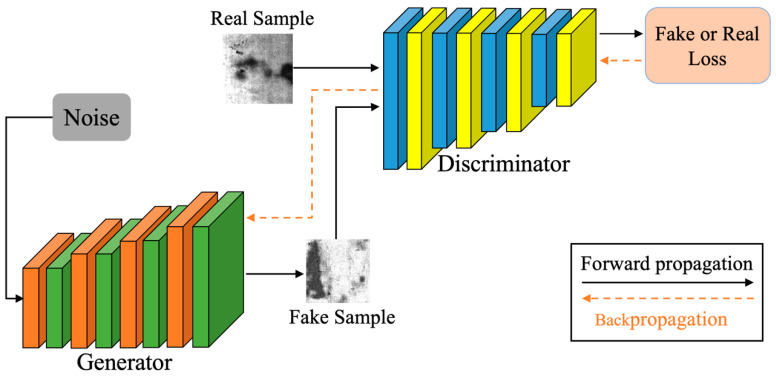
The structure of the EDCGAN.

In the EDCGAN we proposed, only the ELU activation function is introduced in the discriminative layer, while that in the generation layer remains unchanged. Because ELU retains the calculation of input less than 0, the calculation time will be slightly longer during forward propagation and backpropagation. Suppose it is used in large quantities in the discriminant network and the generation network at the same time. In that case, the difficulty of network optimization will further be increased. The discriminative network usually dominates the DCGAN training, and the generative network updates itself according to the discriminative network. Through adversarial training, the change in the high-dimensional mapping ability of the discriminative network will also be transmitted and adjusted to the generative network. Therefore, to obtain richer features without increasing the computational complexity, this paper only uses the ELU function in the discriminant network. Its structure of EDCGAN can be seen in flow Figure 3.

Compared with the DCGAN model, the EDCGAN based on ELU has the following characteristics: (1) It makes full use of the self-normalization characteristics of ELU, omits the batch normalization layer, and reduces the special normalization calculation of parameters. (2) Only use ELU in the discriminant network and use ELU to calculate and extract richer high-dimensional features for data less than 0. (3) To cooperate with the characteristics of the self-normalized activation function, the normalization feature is used in the network, which not only prevents the over-fitting problem associated with the initial training of the discriminant but also speeds up the update speed of the network parameters. 

### 3.2. DropOut

**Figure 4:**
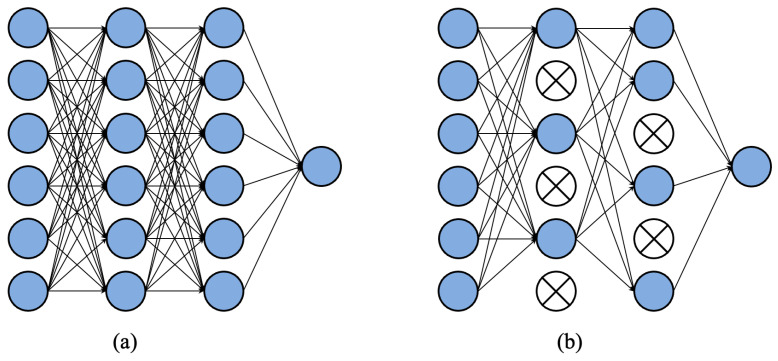
The structure flow chart of DropOut. (**a**) Is the standard neural network structure. In (**b**), some neurons are randomly deleted using dropout.

In the process of model training, using DropOut can discard some neural units, thereby reducing training complexity.. For different batches of input samples, because the discarded neural units are random each time, it is equivalent to training a different network for each batch. Using DropOut can reduce the computational parameters during training and overfitting. The principle of DropOut is shown in Figure 4. In the fully connected network layer, every time the network parameters are updated, some neurons are randomly discarded so that the complexity of the network and the amount of parameter calculation are obviously decreased. 

In the ordinary DCGAN structure, DropOut is not used; because the BN layer is included in those network layers, adding DropOut will complicate the calculation. The EDCGAN does not use the BN layer. The discriminant network and the generation network are mutually influenced, and they keep changing dynamically and alternately during the training process; there is no fixed number of iterations. Therefore, the introduction of DropOut can not only alleviate the over-fitting issue associated with the easy training of the discriminant network but also accelerate the speed of network updates.

Using DropOut is usually to set an activation value x; and set its probability to 1−q, where 0<q≤1. This kind of DropOut is very suitable for correcting linear units, such as ReLU activation functions. However, for the ELU activation function, since the scaled linear exponential unit is used, a parameterized a_Dropout method can be used to obtain better results. First, make $\alpha =\underset{x\to 0}{\min}ELu\left(x\right)=-\lambda x.$ The value of λ is usually a number from 0 to 1, we set it as 0.5.

(6) The mean equation is: E(xd+σ(1−d))=qμ+(1−q)σ

(7) $$\mathrm{The}\;\mathrm{variance}\;\mathrm{formula}\;\mathrm{is}:\;V_{\alpha r}(xd+\sigma (1-d))=q(\left(1-q\right){\left(\sigma -\mu \right)}^{2}+\nu )$$

To keep the mean and variance unchanged after adding the parameter σ, it is necessary to use the parameters α and b to perform the affine transformation on the equation. The transformed mean and variance formulas are:

$$E(\alpha (xd+\sigma \left(1-d\right))+b)=\mu$$

(8) $$V_{\alpha r}(\alpha (xd+\sigma \left(1-d\right))+b)=\nu$$

After using DropOut, the calculation process formula of the previous item of the hidden layer parameter $\omega_{i}$ and $b_{i}$ of the ELU-based network is:

$$r_{j}^{l}\sim Bernoulli\left(q\right)$$

(9) $${\tilde{y}}^{l}=r^{\left(l\right)}\times y^{\left(l\right)}\;z_{i}^{\left(l+1\right)}+\omega_{i}^{\left(l+1\right)}{\tilde{y}}^{l}+b_{i}^{\left(l+1\right)}\;y_{i}^{\left(l+1\right)}=f\left(z_{i}^{\left(l+1\right)}\right)$$

In Formula (9), l represents the lth layer of the neural network, $z_{i}^{\left(l\right)}$ is the input of the l layer, $y_{i}^{l}$ represents the output of the l laye. For any layer l, $\gamma^{\left(l\right)}$ is a vector of independent Bernoulli random variables, each element in this vector is usually set to probability $\left(1-q\right)$ and multiplied by the output $y_{i}^{\left(l\right)}$, thereby reducing the number of computations involved in the training process. Finally, by using the DropOut structure, the calculation amount of it is decreased.

### 3.3. Motivation for Architecture Design

The framework we designed mainly considers two practical problems: (1) In the industrial field, the number of data sets of metal surface defects is difficult to obtain, which makes training difficult. (2) In the manufacturing environment, there is usually no powerful computing equipment, so the selection of the classification algorithm is very important. 

We propose to use GAN models to generate more defect datasets to address the problem of insufficient datasets in the industrial domain. GAN is an adversarial game thinking (see Figure 1), we chose DCGAN, which is more suitable for generating spiced spices, as the backbone network (see Figure 3), and found that replacing the activation function with ELU can effectively improve the performance of the generative model. In this process, we used DropOut tricks to improve the training speed of the network. In the classification method, we use MobileNet V2 as the backbone network, mainly because it has a faster running speed while ensuring good classification results. In the next section, we demonstrate the feasibility of the proposed method through experiments. In the next section, the effectiveness and superiority of the method will be demonstrated through experiments.

## 4. Experiments and Results

This part showed the experimental results of our method used for the surface defect classification of steels. We first tested the classification results of MobileNet V2 under different proportions of the original samples and finally compared different classification methods. The hardware platform of all experiments is RTX3080Ti 12G graphics card with the Intel i9-13900K processor. The operating system is Linux, and the Scientific Computing Package Framework is Pytorch (1.13.0).

### 4.1. NEU-CLS Dataset

**Figure 5:**
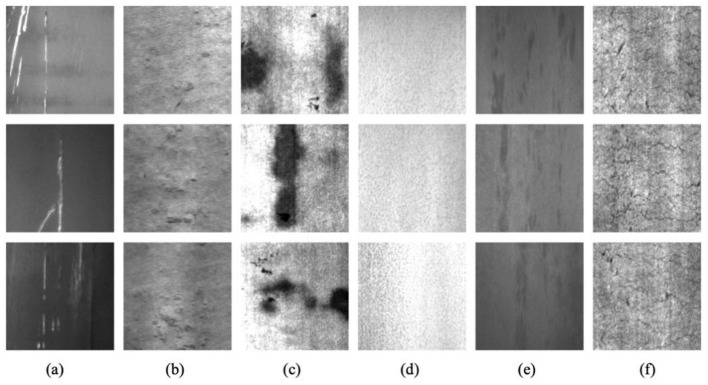
Examples of defect samples in the NEU-CLS. (**a**) Sc. (**b**) Rs. (**c**) Pa. (**d**) PS. (**e**) In. (**f**) Cr.

NEU-CLS (http://faculty.neu.edu.cn/songkechen/zh_CN/zdylm/263270/list/index.htm accessed on 10 September 2022) is a defect dataset provided by Northeastern University [25]. All image samples in NEU-CLS are captured by the CCD camera on hot-rolled steel plates surface and are manually filtered and cropped to obtain the same size. NEU-CLS contains six common representative steel surface defects: scratches (Sc), rolled-in scale (RS), patches (Pa), pitted surface (PS), inclusion (In), and crazing (Cr). There are a total of 1800 defect samples: 6 different defect types, each with 300 samples and 200 × 200 in size, as the result in Figure 5. For the classification task of NEU-CLS, there are two problems, that is, different types of defects have a high degree of similarity in appearance; the images of defects are affected by illumination changes and material deformation. 

In the experimental part of this paper, we split the NEU-CLS dataset into training and test sets with a ratio of 7: 3. Finally, we obtain a total of 1260 training samples per class and 540 testing samples in total. Compared with other datasets with massive samples, such as CIFAR [26], ImageNet [27], Caltech 101 [28], and PASCAL VOC [29], the NEU-CLS is extremely small-scale. The number of samples in the NEU-CLS dataset is very small and cannot effectively train the network model. It is for this reason that we try to use the EDCGAN to generate more training samples to meet the learning needs of the deep learning model. The mini-batch size is 128 for each input of the model. We train EDCGAN using the MBGD [30] algorithm with exponential decay parameters *β* 1 and *β* 2 set to 0.8 and 0.99, respectively. EDCGAN was trained for 800 epochs with a learning rate of 0.0001. For MobileNet V2, we use the stochastic gradient descent (SGD) [31] algorithm with a learning rate of 0.001. We train the model for 300 epochs in the initial step, then 600 epochs in the retraining step.

### 4.2. Data Augmentation Based on EDCGAN

For the generative model, first, we fed a 100-dimensional random noise vector and then reshaped it to the size of 4×4×1024 with a linear function. After deconvolution, the size becomes 8×8×512; then, perform batch normalization (BN) on the value of all tensors, and use ERelu to activate the function. The structure of the entire generative model collectively undergoes six deconvolution processes, each with a kernel size of 3×3 and a stride of 2. In the end, the generator model generates a 256×256 size image. Meanwhile, the discriminator receives the generated and real samples as input. Similar to the generator, the discriminator has six convolutional layers for processing tensors. The size of these layers is also 3 × 3, and the stride is 2. Since BN is already included in the ERELU activation function, there is no need to add a BN function. Finally, the discriminator outputs a discrete distribution probability for each input sample to judge if the input sample is a real image. Through training, eventually, the discriminator cannot identify if the input image is from the generator or the real sample. At this time, we can think that the model has reached the best performance. The network architecture of the DCGAN model is shown in Table 1.

**Figure 6:**
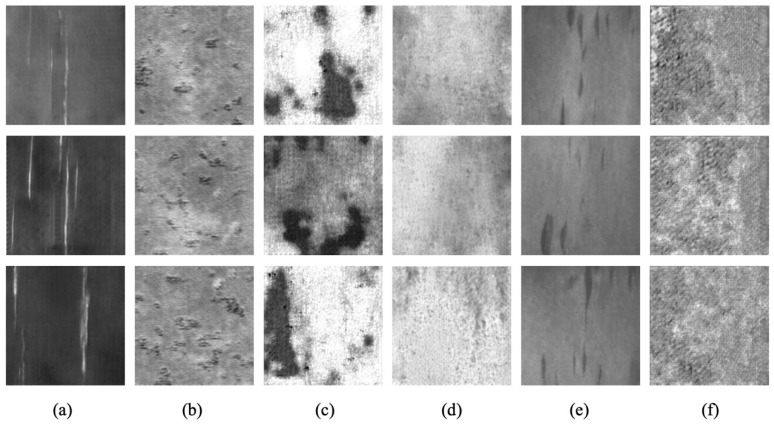
Examples of defect samples generated by EDCGAN. (**a**) Sc. (**b**) Rs. (**c**) Pa. (**d**) Ps. (**e**) In. (**f**) Cr.

In order to obtain more training samples, we use EDCGAN to obtain abundant fake defect samples to train the model. We send a total number of 1260 real defect samples into EDCGAN and, finally, gain 8000 fake defect sample images; all the sizes are 224×224; some of the generated samples are shown in Figure 6. According to the result in Figure 6, for most types, EDCGAN has a good generation effect, such as Sc, Rs, Pa, and In. For these kinds of generated samples, it is difficult to distinguish them from real sample images or not, even if human eyes observe them. However, not all types generate good results. For Pa, the generated images have a certain gap with the real sample images; especially for Ps, the generated samples are significantly different from the original images. The improvement of classification accuracy is limited, which is also proven by the following experimental results.

### 4.3. Classification Results under Different Numbers of GAN Samples

To analyze the influence of the generated samples on classification results, we use different proportions of the generated dataset in the training dataset. According to the different proportions of the original samples, this paper divides the dataset into six types. The proportion of the original samples is from high to low. In the dataset containing raw data samples, up to 100% of the raw samples are included and then decrease initially from 70%, 50%, and 30% to 20%. We also tested a dataset entirely generated by DCGAN that did not contain any original samples. We test the classification accuracy of MobileNet V2 under different proportions of the dataset to obtain the results, which can be seen in Table 2. We use the average precision (*AP*) to evaluate the experimental results, which is a good compromise between two important detection metrics. These metrics can be expressed as follows:

(10) $$Precision=\frac{TP}{TP+FP}$$

(11) $$Recall=\frac{TP}{TP+FN}$$

(12) $$AP=\frac{Precision+Recall}{2}$$

In which *TP*, *FP*, and *FN* refer to the true positives; and false positives/negatives, respectively. 

According to the result in Table 2, under the condition of the training set, containing the original image, the classification accuracy is the lowest. With the addition of DCGAN to generate sample images, the detection results are improving. When DCGAN sample images account for 80% of the training set, the classification result reaches the highest point. At the same time, it can be noticed that the accuracy of In. does not increase linearly with the increase in the dataset. When the training samples increase to 6300, the resolution accuracy decreases slightly. This may be because DCGAN is not very good at generating In. When the DCGAN sample data dataset is completely used, the accuracy of classification decreases and the sample images generated by DCGAN can be delayed describing only a part of the features of the original dataset; they cannot fully express all the feature data of the original samples. 

To test the classification results more abundantly, we selected several other classification algorithms for comparative experiments. Specifically, the methods of Zhou [32] who applied small CNNs based on VGG-16 [33], and ResNet-19 [34]. In the comparison process, all the above models were reconstructed based on the Pytorch package. We use the average accuracy rate of the six classification results as the evaluation index and tested the results when no generated samples were added, when 80% generated samples were included, and when only generated samples were included. Table 3 shows the results of different classification models.

From the table above, we can find that the fully supervised learning method is highly dependent on the original sample, so when the data does not contain the original sample, the accuracy rate is not the lowest, only 90.51%. As the data set increases, the overall classification effect is improved for the other classification methods. When only 20% of the original samples are included, the accuracy of VGG reaches 99.23%, while when only generated samples are included, the accuracy drops slightly. Using the classification method of MobileNet V2, the highest average accuracy rate reached 99.11%, which is slightly lower than ResNet, but considering that the network structure of MobileNet V2 is simpler, it can save training time. Therefore, this paper can obtain the result that MobileNet V2 is more suitable for classification tasks in industrial scenarios.

## 5. Conclusions

This study constructed a method that depended on EDCGAN and MobileNet V2 to classify steel surface defects. Considering the lack of original training samples, we propose an EDCGAN method to generate abundant new training samples. The generated samples are not transformations of the original samples, so it can better improve the diversity of the training dataset. To confirm the validity of generated samples, we use the NEU-CLS defect dataset as the experimental object, mix the generated samples with the original samples in different proportions, and then train on the MobileNet V2 model, and use the training results to test and compare the effect of including different proportions of generated samples in the training set on the classification results. Through many experimental comparisons, it can be shown that in the steel surface defect discrimination method, despite the number of original samples being limited, we can still increase the training samples through our method, thereby improving the accuracy. Finally, we compared the evaluation accuracy results of different algorithms. According to the result that both VGG and MobileNet V2 have higher accuracy, and MobileNet V2 has a simpler network structure, it is more suitable for steel defect classification tasks in industrial production.

## Figures and Tables

**Table 1:** The network framework of the EDCGAN.

Generator	Type	Size/Channel	Size/Stride	Discriminator	Type	Size/Channel	Size/Stride
D1	deconv	8 × 8 × 512	3×3/2	C1	conv	256 × 256 × 3	3×3/2
E1	EReLu	-	-		EReLu	-	-
D2	deconv	16 × 16 × 256	3×3/2	C2	conv	128 × 128 × 32	3×3/2
E2	EReLu	-	-		EReLu	-	-
D3	deconv	32 × 32 × 128	3×3/2	C3	conv	64 × 64 × 64	3×3/2
E3	EReLU	-	-		EReLu	-	-
D4	deconv	64 × 64 × 64	3×3/2	C4	conv	32 × 32 × 128	3×3/2
E4	EReLu	-	-		EReLu	-	-
D5	deconv	128 × 128 × 32	3×3/2	C5	conv	16 × 16 × 256	3×3/2
E5	EReLu	-	-		EReLu	-	-
D6	deconv	256 × 256 × 3	3×3/2	C6	conv	8 × 8 × 512	3×3/2
E6	tanh	-	-		EReLu	-	-

**Table 2:** Classification results from various numbers of samples.

Training Set Original (num.)	Overall Accuracy (%)					
	Sc	Rs	Pa	Ps	In	Cr
1260(100%)	97.42	95.64	96.45	95.26	94.27	95.41
1680 (75%)	97.82	97.36	98.51	93.65	95.68	97.68
2520 (50%)	98.75	98.32	98.72	96.81	95.78	98.04
4200 (30%)	99.52	99.02	98.45	98.92	96.04	98.45
6300(20%)	99.94	99.67	99.05	98.05	98.74	99.25
8000(0%)	98.21	98.58	98.34	97.86	97.65	96.88

**Table 3:** Comparisons of different defect classifiers.

		Average Accuracy(%)	
Methods	org. (100%)	org. (20%)	org. (0%)
Fully supervised learning [32]	94.14	95.21	90.51
VGG-16 [33]	95.81	98.63	96.95
ResNet-19 [34]	97.62	99.23	98.92
MobileNet V2	95.74	99.11	97.92

## Data Availability

Not applicable.

## References

[1] Essid O., Laga H., Samir C. (2018). Automatic detection and classification of manufacturing defects in metal boxes using deep neural networks. PLoS ONE.

[2] Luo Q., Fang X., Sun Y., Simpson O. (2019). Surface defect classification for hot-rolled steel strips by selectively dominant local binary patterns. IEEE Access.

[3] Luo Q., Fang X., Liu L., Yang C., Sun Y. (2020). Automated visual defect detection for flat steel surface: A survey. IEEE Trans. Instrum. Meas..

[4] Ahonen T., Hadid A., Pietikäinen M. Face recognition with local binary patterns. Proceedings of the 8th European Conference on Computer Vision.

[5] Lienhart R., Maydt J. An extended set of haar-like features for rapid object detection. Proceedings of the International Conference on Image Processing.

[6] Dalal N., Triggs B. Histograms of oriented gradients for human detection. Proceedings of the 2005 IEEE Computer Society 131 Conference on Computer Vision and Pattern Recognition (CVPR’05).

[7] Aghdam S.R., Amid E., Imani M.F. A fast method of steel surface defect detection using decision trees applied to LBP based 133 features. Proceedings of the 2012 7th IEEE Conference on Industrial Electronics and Applications (ICIEA).

[8] Ghorai S., Mukherjee A., Gangadaran M., Dutta K.P. (2012). Automatic defect detection on hot-rolled flat steel products. IEEE Trans. Instrum. Meas..

[9] Shumin D., Zhoufeng L., Chunlei L. AdaBoost learning for fabric defect detection based on HOG and SVM. Proceedings of the 2011 International Conference on Multimedia Technology.

[10] Shrestha A., Mahmood A. (2019). Review of deep learning algorithms and architectures. IEEE Access.

[11] Fu G., Sun P., Zhu W., Yang J., Cao Y., Yang Y.M., Cao Y. (2019). A deep-learning-based approach for fast and robust steel surface defects classification. Opt. Lasers Eng..

[12] Huang Z., Wu J., Xie F. (2021). Automatic recognition of surface defects for hot-rolled steel strip based on deep attention residual convolutional neural network. Mater. Lett..

[13] Li J., Su Z., Geng J., Yin Y. (2018). Real-time detection of steel strip surface defects based on improved yolo detection network. IFAC-Pap..

[14] Tao X., Zhang D., Ma W., Liu X., Xu D. (2018). Automatic metallic surface defect detection and recognition with convolutional neural networks. Appl. Sci..

[15] He Y., Song K., Meng Q., Yan Y. (2019). An end-to-end steel surface defect detection approach via fusing multiple hierarchical features. IEEE Trans. Instrum. Meas..

[16] Li M., Wang H., Wan Z. (2022). Surface defect detection of steel strips based on improved YOLOv4. Comput. Electr. Eng..

[17] Kim Y., Kwak G.H., Lee K.D., Na S.I., Park C.W., Park N.W. (2018). Performance evaluation of machine learning and deep learning 153 algorithms in crop classification: Impact of hyper-parameters and training sample size. Korean J. Remote Sens..

[18] Shorten C., Khoshgoftaar T.M. (2019). A survey on image data augmentation for deep learning. J. Big Data.

[19] Chlap P., Min H., Vandenberg N., Dowling J., Holloway L., Haworth A. (2021). A review of medical image data augmentation techniques for deep learning applications. J. Med. Imaging Radiat. Oncol..

[20] Simonyan K., Zisserman A. (2014). Very deep convolutional networks for large-scale image recognition. arXiv.

[21] Krizhevsky A., Sutskever I., Hinton G.E. (2017). Imagenet classification with deep convolutional neural networks. Commun. ACM.

[22] He K., Zhang X., Ren S., Sun J. Deep residual learning for image recognition. Proceedings of the 2016 IEEE Conference on Computer Vision and Pattern Recognition (CVPR).

[23] Howard A.G., Zhu M., Chen B., Kalenichenko D., Wang W., Weyand T., Andreetto M., Adam H. (2017). Mobilenets: Efficient convolutional neural networks for mobile vision applications. arXiv.

[24] Hu J., Shen L., Sun G. Squeeze-and-excitation networks. Proceedings of the 2018 IEEE/CVF Conference on Computer Vision and Pattern Recognition.

[25] Bao Y., Song K., Liu J., Wang Y., Yan Y., Yu H., Li X. (2021). Triplet-graph reasoning network for few-shot metal generic surface defect segmentation. IEEE Trans. Instrum. Meas..

[26] Sharma N., Jain V., Mishra A. (2018). An analysis of convolutional neural networks for image classification. Procedia Comput. Sci..

[27] Deng J., Dong W., Socher R., Li L.J., Li K., Li F.F. Imagenet: A large-scale hierarchical image database. Proceedings of the 2009 IEEE Conference on Computer Vision and Pattern Recognition.

[28] Li F.F., Andreeto M., Ranzato M., Perona P. Caltech 101 (1.0) [Data Set]. CaltechDATA.

[29] Everingham M., Gool L.V., Williams C.K.I., Winn J., Zisserman A. (2010). The pascal visual object classes (voc) challenge. Int. J. Comput. Vision.

[30] Ruder S. (2016). An overview of gradient descent optimization algorithms. arXiv.

[31] Bottou L. (2012). Stochastic gradient descent tricks. Lect. Notes Comput. Sci..

[32] Zhou S., Chen Y., Zhang D., Xie J., Zhou Y. (2017). Classification of surface defects on steel sheet using convolutional neural networks. Mater. Technol..

[33] Sengupta A., Ye Y., Wang R., Liu C., Roy K. (2019). Going deeper in spiking neural networks: VGG and residual architectures. Front. Neurosci..

[34] Chen W., Gao Y., Gao L., Li X. (2018). A new ensemble approach based on deep convolutional neural networks for steel surface defect classification. Procedia CIRP.

